# The “Kickstand Rod” Technique for Coronal Imbalance in Patients With Spinal Deformity: A Case Report With Review of Literature

**DOI:** 10.7759/cureus.11876

**Published:** 2020-12-03

**Authors:** Brian Fiani, Ryan M Jarrah

**Affiliations:** 1 Neurosurgery, Desert Regional Medical Center, Palm Springs, USA; 2 Neurosurgery, Mayo Clinic, Rochester, USA

**Keywords:** coronal imbalance, spinal deformity, kickstand rod, deformity correction, spinal instrumentation, posterior long segment fusion, posterior spinal fixation and fusion, lumbar-fusion, sagittal balance, scoliosis

## Abstract

Coronal imbalance is a type of spinal deformity with deviation from midline in the coronal plane. It is challenging to correct even in the hands of experienced spine surgeons. Many conventional techniques lead to unsuccessful results or complications. However, the incorporation of “kickstand rod” (KR) instrumentation is now understood to provide a more supported coronal correction and improve spinal deformities. Sometimes it can be used to provide additional spinal support in instances where spinal fusion has already occurred. The KR is placed from a posterior approach along the lateral spine from lumbar spine to ilium and exerts distraction forces that counteract misaligned spinal segments. Our objective is to present a clinical case example with a brief review of literature. Herein, we present a case of a 62-year-old male with the development of significant coronal imbalance following his posterior lumbosacral instrumentation and fusion 11 years prior to presentation. KR supplementation to his hardware improved his functional outcome significantly. Further, we provide a literature review of the surgical characteristics, indications, and functional outcomes of KR instrumentation. A term search of “kickstand rod” was performed in PubMed, and relevant English language publications were included. The literature search yielded only six publications. A total of 45 patients across three studies were assessed. A mean postoperative coronal balance magnitude of 26.83 mm was calculated compared to the preoperative coronal magnitude of 64.16 mm. Results also showed only four cases of intraoperative or postoperative complications. Moreover, the presented case reported successful KR implementation without any intraoperative complications. KR instrumentation is a safe and effective technique for coronal imbalance correction. The results show favorable outcomes in terms of coronal adjustment and low complication rates. Nevertheless, we caution the fact that further studies are warranted with long-term follow-ups.

## Introduction

Coronal imbalance (CI) is one of the most frequent, yet misunderstood, spinal deformities [1]. CI is defined as a noticeable lateral displacement of the C7 plumbline (C7PL) from the mid-sacrum and typically occurs secondary to scoliosis or acquired reasons [2]. The displacement can cause significant adult and pediatric spinal deformity with debilitating symptoms that lead to disability and immobility. With limited compensatory mechanisms, CI correction is paramount, yet challenging surgically. Most CI patients tend to have multiple comorbidities, previous spinal operations, superimposed degenerative changes, and rigid spinal curvatures, all factors that complicate the surgical process [3]. Pedicle subtraction osteotomy (PSO) has been described as a treatment method; however, its incidence of infection, high rate of neurological deficits, high operative blood loss volume, and risk of pseudoarthrosis (PA) development make it somewhat non-ideal for patients and surgeons alike [4]. Moreover, due to coronal imbalances being associated with sagittal misalignments, surgical biplanar corrective maneuvers may be performed, yet also present its own challenges along with a high rate of surgical complications [3].

A recent technique has been developed to surgically improve coronal balance. Kickstand rod (KR) implantation is a posterior approach spine surgery technique that involves positioning a rod onto the side of the concave CI to allow for distraction forces to counteract the misalignments and ultimately lead to a more normal coronal balance [5]. The rod is held in place by pedicle screws and an iliac screw. The technique presents secondary benefits by offloading mechanical stress and bolstering primary instrumentation, ultimately reducing occurrence of rod-fracture, hardware failure, or PA [5]. However, aside from these clinical benefits, there are currently very limited publications involving cases or studies that describe the outcomes of KR instrumentation for CI. In fact, a PubMed term search of “kickstand rod” only yields six publications, and those were from 2018 to 2020 identifying the recent usage and interest in this technique.

Herein, we present a case of an adult male with significant thoracolumbar CI and inability to stand unassisted or ambulate secondarily acquired from prior spine surgery. After the KR technique was successfully performed, he was ambulating 275 feet on postoperative day 1. Additionally, a literature review is performed to discuss the works of previous studies that investigate this novel technique in order to provide a comprehensive overview of KR’s surgical characteristics, indications, and functional outcomes.

## Case presentation

We present a 62-year-old Hispanic male who presented to our institution with a chief complaint of low back pain. He had a past surgical history significant for lumbar 3 (L3)-sacral 1 (S1) posterior lumbar instrumentation and fusion status-post traumatic fall, which had caused him to have compression fractures and kyphotic deformity. That surgery was performed in 2009 at outside institution. He did not seek postoperative follow-up. At current presentation, the patient stated that he recently fell approximately three weeks prior. He started having a new onset of severe back pain, and he also endorsed some associated radiculopathy in the S1 distribution on the left side greater than right side. He admitted that he had been using a walker at baseline since his prior surgery but then progressed to needing a wheelchair, then immobile after the fall three weeks prior. He denied any bowel or bladder incontinence; however, he did endorse not making it to the bathroom in time because he is not ambulating. He denied any intravenous drug use or fevers. His motor strength examination was significant for weakness in his lower extremities as follows: right ankle dorsiflexion 2/5, right ankle plantarflexion 3/5, and left ankle dorsiflexion 3/5. Whether the weakness was from neurological source or from disuse was not defined. Sensation and rectal exams were unremarkable. On physical therapy assessment, the patient was unable to ambulate.

**Figure 1:**
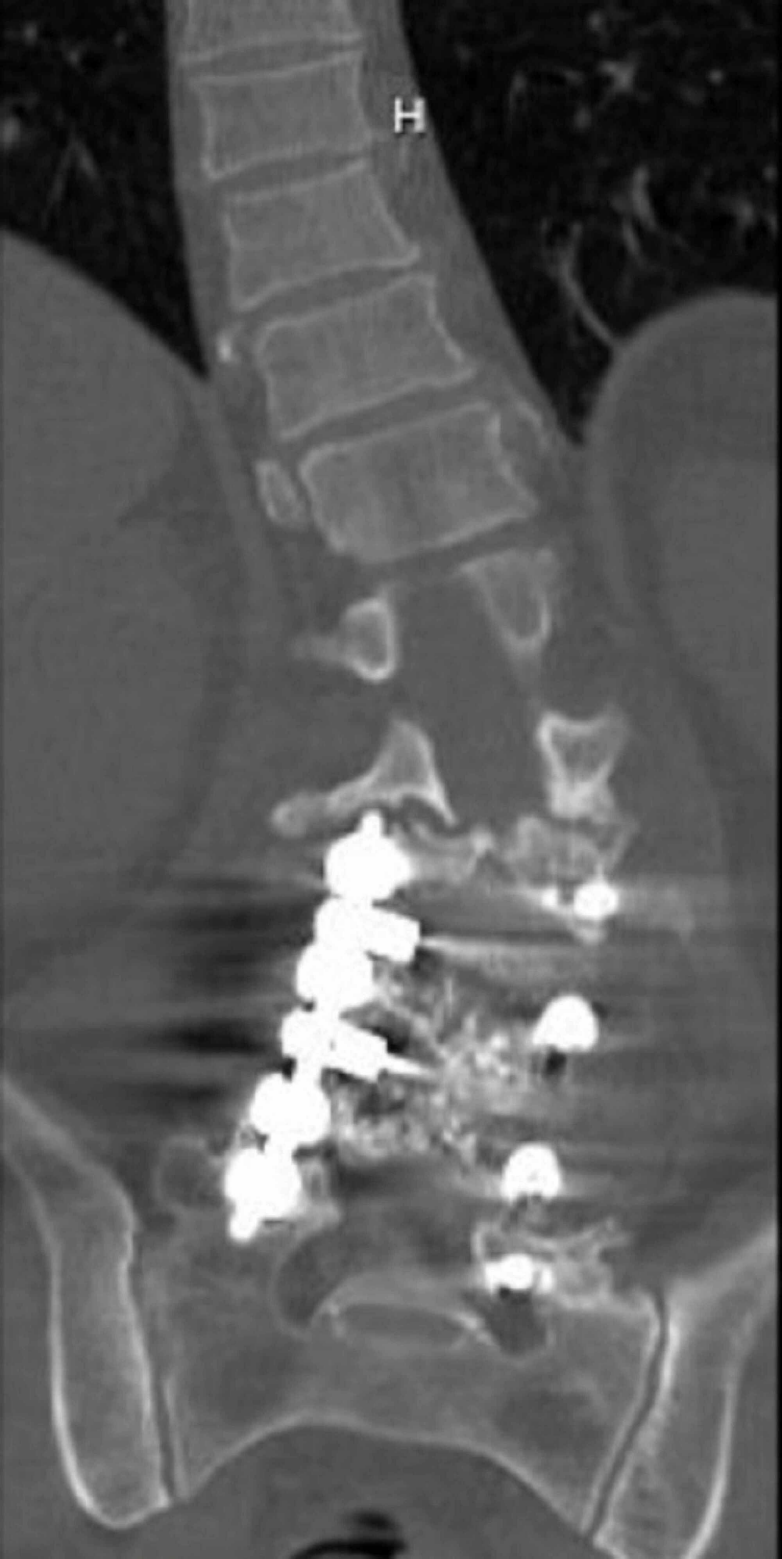
Preoperative CT of the lumbar spine, coronal view, displays the curvature of the patient's lumbar spine and identifies the existing hardware from prior surgery. CT, computed tomography.

**Figure 2:**
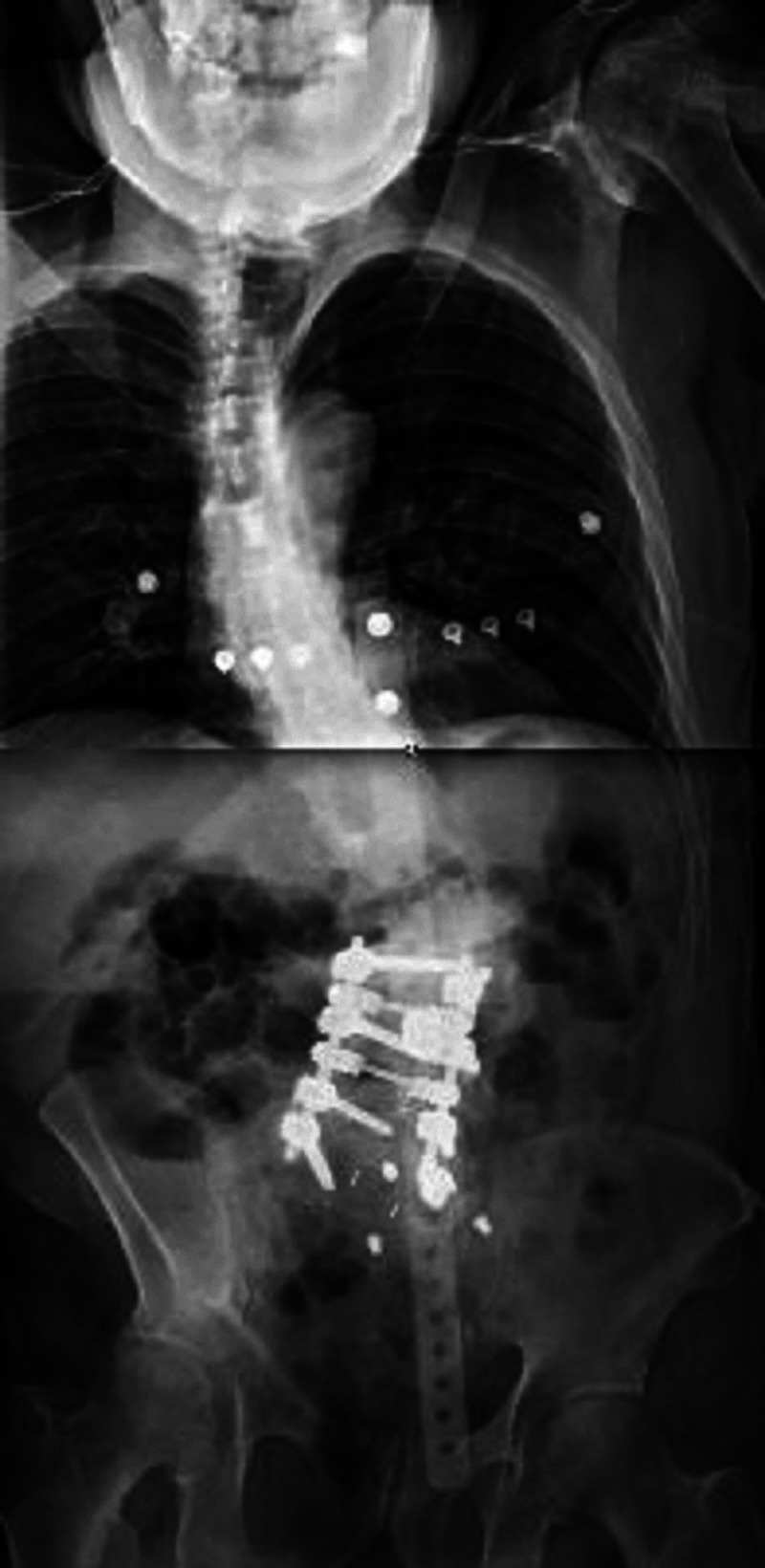
Preoperative standing upright scoliosis x-ray films show the severe S-shaped scoliotic curvature of the patient’s thoracolumbar spine with coronal imbalance prior to kickstand rod surgery.

**Figure 3:**
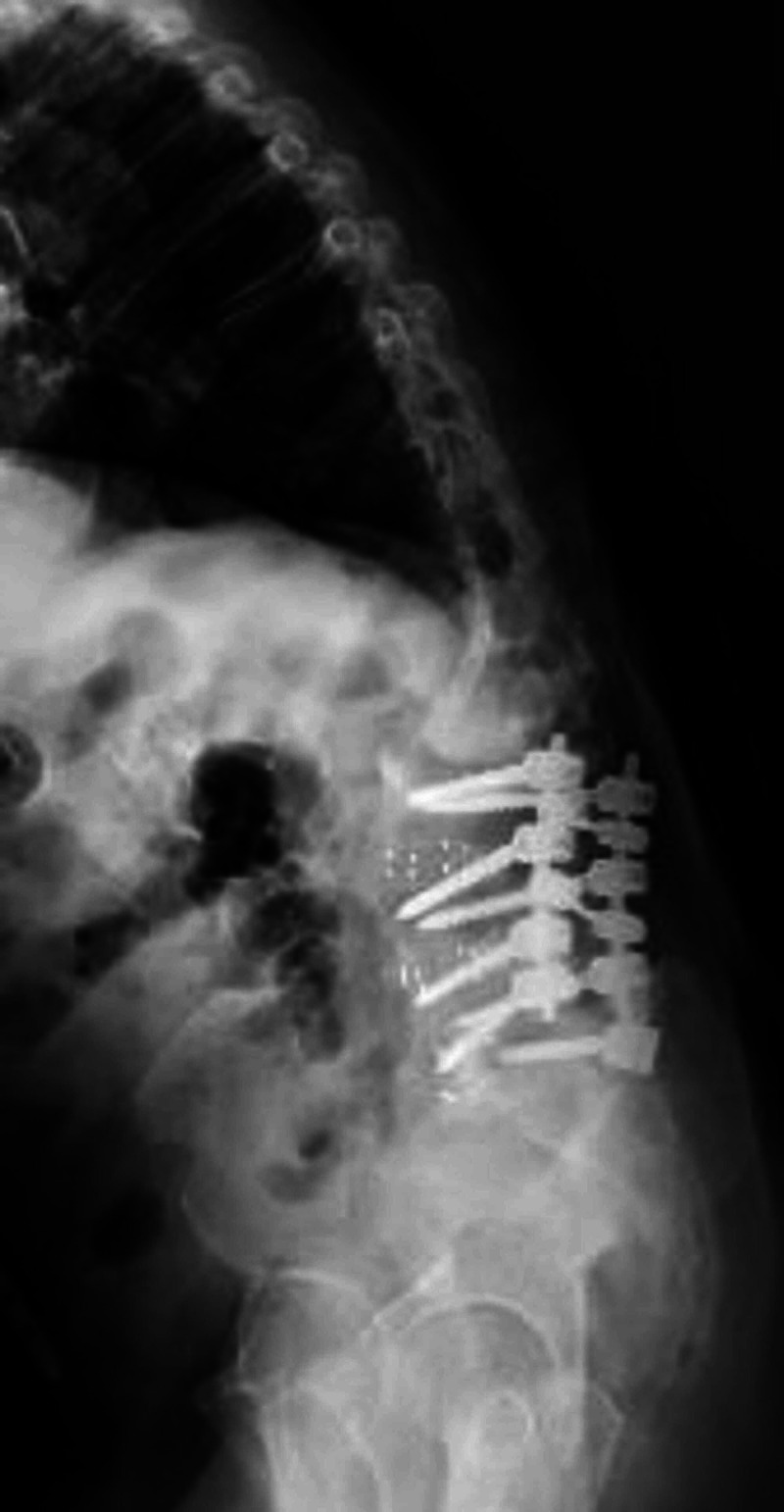
Preoperative standing upright scoliosis x-ray films shows the kyphotic deformity with patient’s inability to stand upright prior to kickstand rod surgery.

Magnetic resonance imaging (MRI) of the lumbar spine showed findings of increased edematous signal at the lumbar 2 (L2)-lumbar 3 (L3) vertebral level. The edematous signal was likely from endplate erosive changes. There was grade 1 retrolisthesis of L2 on L3 with mild to moderate generalized disc bulging and bilateral facet arthropathy with severe central canal and bilateral neural foraminal narrowing, and there was grade 2 anterolisthesis of L5 (lumbar 5) on S1. Computed tomography (CT) of the lumbar spine showed left convex-right concave curvature of the lumbar spine (Figure 1). Standing upright scoliosis films showed moderate to severe S-shaped scoliotic curvature of the thoracolumbar spine with CI (Figures 2, 3). His CI measurement was 64 mm of lateral displacement of the C7 plumbline (C7PL) from the mid-sacrum. Stable erosive changes the right aspect of the L2-L3 endplate at the apex of severe left convex lumbar scoliotic curvature.

**Figure 4:**
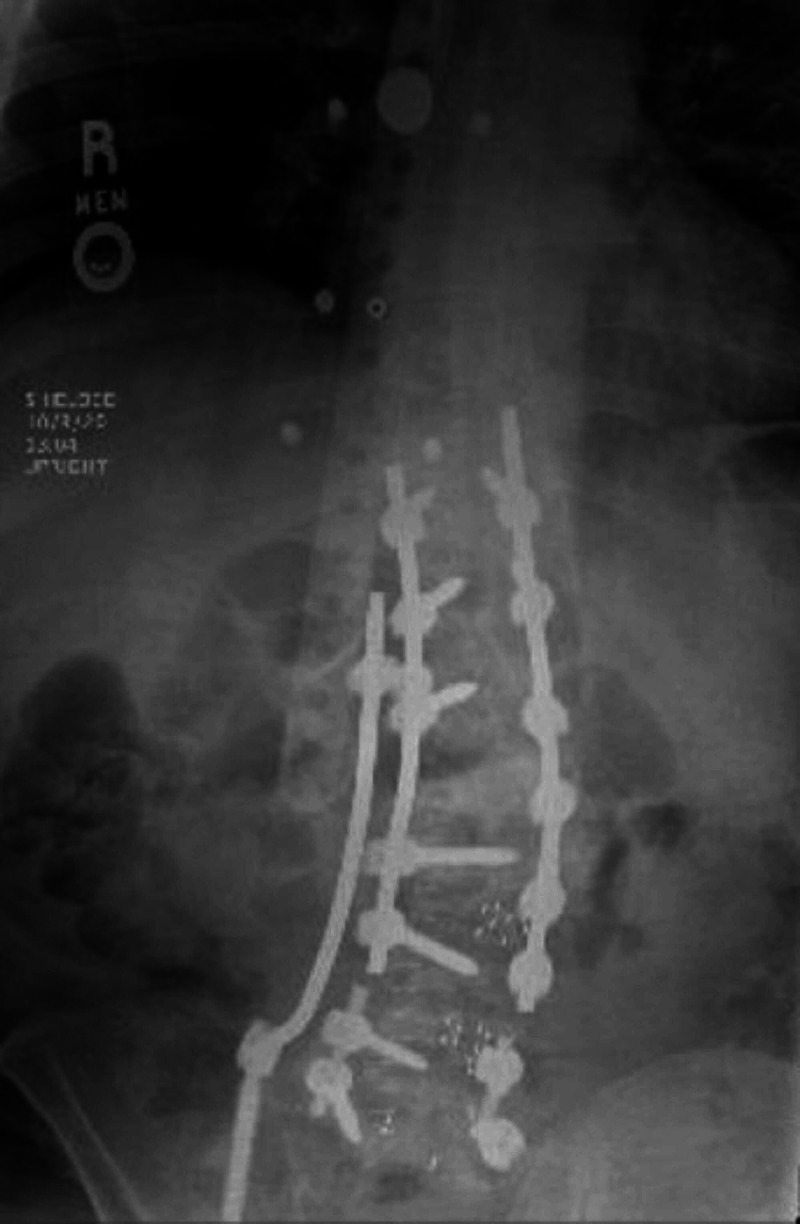
Postoperative standing upright lumbar spine x-rays defining the kickstand rod placement along the patient’s lumbar-iliac junction.

It was determined that the ideal surgical procedure was a posterior approach to place a right-sided KR from lower thoracic spine to ilium. The prior surgical fusion was complete, and therefore the goal was not to adjust his fused imbalance but rather to provide structural support for his spinal malalignment. First, a hardware extension was performed from T11 (thoracic 11) to L2 with posterior segmental instrumentation and fusion. Second, the rods were cut at L4 (lumbar four)-L5 to avoid additional dissection of screws lower than L4-L5 interbody in order to place longer rods. Lastly, an 8.5 mm x 60 mm right iliac screw with 5.5 mm cobalt chrome KR was placed. Preoperatively, the patient ambulated 0 feet upon physical therapy assessment. On the first postoperative day, the patient ambulated 275 feet with a front wheeled walker. Within one week, he ambulated 900 feet with the physical therapy team. Standing upright thoracolumbar x-rays postoperatively were taken identifying the KR instrumentation and improved appearance of his CI (Figure 4). However, patient eloped from the hospital and was lost to follow-up; therefore, new scoliosis standing upright x-rays for new measurement of CI were unobtainable for comparison.

## Discussion

Technique

The following is a brief description of the surgical technique; it differs from our aforementioned stated technique due to our patient already having prior segmental pedicle screw instrumentation and fusion surgery. The surgical placement of a KR generally involves the usage of large-diameter screws, a rigid rod, sometimes rod-to-rod connectors, and a reliance on the mechanical stability of the pelvis in order to achieve clinical success. Using an all-posterior approach under general anesthesia, subperiosteal exposure of the spine is achieved by carefully placing the patient prone on a supportive bed frame with attempt to maintain natural alignment, leaving the abdomen and axilla free [6]. Decompressions, facetectomies, and posterior column osteotomies are subsequently performed to relieve neural pressures, mobilize the spinal region, and correct fractional curves [6]. Pedicle screws are then inserted segmentally, as these titanium screws act as anchor points that can be later connected to rods.

The technique is followed with attention on pelvic fixation as either sacral-alar-iliac screws or iliac screws reinforce this action [3]. Focusing on the later, the iliac screw is inserted on the side of the coronal malalignment. When placing bilateral screws and rods, a full-length primary rod is then placed ipsilaterally to the misalignment, while a short-temporary rod is placed on the contralateral side spanning across the lower fractional spinal segments [3]. The screws of this short-temporary rod are subsequently tightened to maintain an inward curvature of the spine during kickstand distraction [3]. The KR is then implemented along the junction levels and on the side of the CI [3,5]. The KR is subsequently secured using a rod-to-rod connector [3]. The screw caps on the primary rod are subsequently unscrewed, and using the iliac wing for support, distraction forces will be exerted. These distraction forces will then generate a powerful corrective torque to decrease the angle of the lumbar major curve and lead to a harmonized coronal alignment [3,5].

Indications

**Figure 5:**
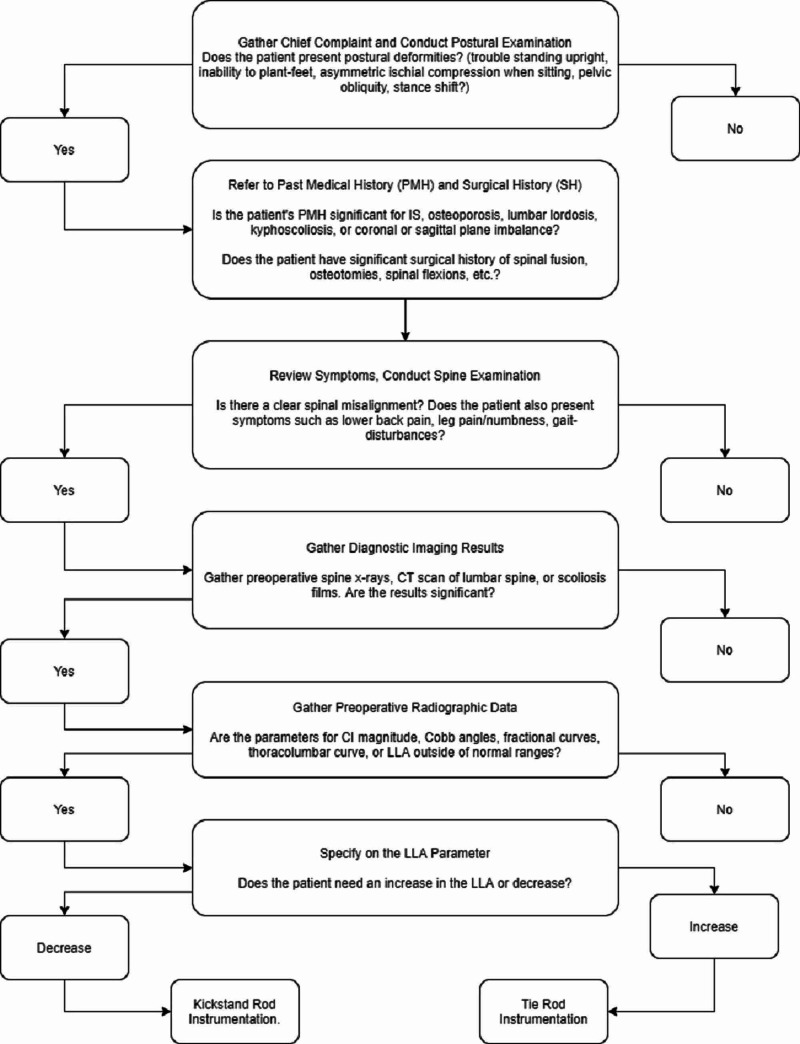
Proposed algorithm for surgeon decision-making. CT, Computed tomography; LLA, lumbar lordosis angle.

There is a paucity of literature incorporating KR application, and there has yet to be a predetermined algorithm that determines whether a patient is a candidate for KR instrumentation. Our authors propose the algorithm in Figure 5. Currently, a surgeon's decision to implement KR is either made preoperatively, if the patient has a significant CI, or intraoperatively after initial instrumentation [6]. Focusing on the former, radiographic data is often used to assess a patient’s malalignment through various parameters [3]. These parameters include magnitude, thoracolumbar curve angle, fractional lumbosacral curve angle, and Cobb coronal angles [3]. However, the parameter that is most commonly used to assess CI is magnitude, with a C7PL lateral displacement of greater than 40 mm from the mid-sacrum being an indicator for severe spinal misalignment [7]. Further studies have also used radiographic sagittal parameters such as pelvic tilt angle, lumbar lordosis angle (LLA), sagittal vertical axis (SVA), and T1 (thoracic 1) tilt angle to assess cases where there is an association between coronal and sagittal imbalance [3]. Table 1 provides some of the normal ranges for these parameters, with significant findings being considered indicators for severe CI and potential KR treatment.

**Table 1:** Normal ranges for radiographic parameters of coronal and sagittal imbalances. The last four listed are sagittal parameters, while the first four are coronal parameters. Significant radiographic parameters are indicators of coronal imbalance and potential kickstand rod instrumentation.

Parameter	Normal Range
Normal coronal balance magnitude	<40 mm [7]
Thoracolumbar curve angle	<10° [8]
Cobb angle	<10° [9]
Fractional lumbosacral curve angle	<10° [10]
Lumbar lordosis angle (LLA)	60° [8]
Pelvic tilt	<20° [8]
Sagittal vertical axis (SVA)	<5 mm [8]
T1 tilt angle	<25° (unestablished) [11]

Aside from the radiographic indicators, CI has pathological and symptomatic indicators that would endorse KR implementation. Patients with idiopathic scoliosis are definitive candidates for this procedure, especially those who have undergone multiple spinal procedures [12]. Moreover, degenerative pathologies, such as osteoporosis, tied with CI indicate a potential for KR implementation because of the technique’s ability to provide a more rigid spinal construct [6]. In addition, sagittal imbalances have also been case indicators for this technique, as overcorrection of positive sagittal imbalances is deemed to benefit from KR application [3]. Patients suffering from severe lumbar lordosis are also candidates for this procedure as the distraction forces from the rod decrease the lumbosacral angle to prevent this deformity. Some of the more symptomatic indicators include persistent back pain that may radiate to the legs, lower leg numbness, trouble standing upright, inability to plant-feet, gait-disturbances, asymmetric ischial compression when sitting, pelvic obliquity, and shifted stance when standing [3]. These profound symptoms can be used to assess the severity of the CI and establish patients for KR instrumentation candidacy.

Outcomes

While the literature on kickstand instrumentation is limited, there is some characterized data that provides the surgical, safety, and functional characteristics of this technique. One study took the pre/postoperative records of 24 spinal deformity patients between the ages of 14 and 73 years who underwent KR instrumentation [6]. The results showed an average preoperative CI magnitude of 63 mm, compared to a postoperative mean CI of 47 mm [6]. This made for an average coronal magnitude change of 16 mm, making the results statistically significant. Moreover, compared to the normal coronal balance value of <40 mm, an average of 47 mm meant that many of the patients are close to achieving normal spinal alignment following the procedure. Furthermore, in a small case series study of four female patients, KR corrective technique was performed on two patients while a similar technique, tie-rod, was done in the other two [13]. In the first KR patient, a 56-year-old woman, she had a preoperative CI magnitude of 55 mm, which was corrected to 35 mm. In addition, she had an LLA of 66° that was altered to 62°, with her procedure resulting in significantly improved clinical outcomes [13]. The second patient, a 68-year old, had a preoperative CI magnitude of 44 mm, which was subsequently corrected to 12 mm. Her LLA was reduced from 58° to 55° while her procedure also had no complications [13]. Her improvement in coronal alignment was evident as her back pain improved substantially along with her ability to walk without aids [13]. With a mean KR correction of 26 mm and no operative complications, the results of this small scale study highlighted the significant impact this operative technique has on improving the functionality and quality of life of patients.

In a further study analyzing 19 adult spinal deformity (ASD) patients, CI was substantially reduced from an average magnitude of 80 mm to a postoperative magnitude of 10 mm [3]. With an average CI magnitude difference of 70 mm, these results show remarkably improved spinal alignments among the sample population [3]. In addition, the major thoracolumbar curve was reduced from an average of 37° to 12°, whereas the angle of fractional curves decreased from an average of 20.1° to 9.6° [3]. With respect to Table 1, these results indicated that KR implementation allowed for these parameters to fall near or within the normal range. This study also revealed appealing results in terms of incidences of complication, as no instrumental complications were observed. Only a single patient suffered from subsequent transient neurological deficits, whereas another suffered from persistent neurological deficits [3]. Two intraoperative complication cases were also recorded, while 16/19 patients were discharged to rehabilitation centers [3]. In addition, an increase in numeric rating scale (NRS) scores among patients was observed between the preoperative and early postoperative follow-ups. Back pain decreased from 7.2 ± 2.0 to 4.2 ± 2.6, while leg pain reduced from 5.0 ± 2.7 to 1.7 ± 2.9, making the findings statistically significant [3]. This study also outlined some of the surgical characteristics as the estimated surgery time was approximated at 5.7 ± 1.2 hours. The surgical blood loss was estimated at 3.0 ± 1.6 liters, and the hospital length of stay was estimated at 10.4 ± 7.5 days [3].

Conclusively from these three studies, a combined average preoperative CI magnitude of 64.16 mm was compared to the combined average postoperative CI magnitude of 26.83 mm (Table 2). This made for a magnitude difference of 37.3 mm with only two cases of postoperative complications from all 45 assessed patients. While much of this data does highly endorse KR instrumentation, it is cautioned that these studies have small sample sizes and only provide early outcomes rather than assessing the longevity of KR instrumentation. Nevertheless, the results of these studies do project the potential that KR has to help improve spinal deformity, and these outcomes should be regarded for the clinical significance.

**Table 2:** Results of three published studies using kickstand-rod implementation for coronal imbalance. With normal coronal imbalance being less than 40 mm, results show that kickstand-rod implementation is an efficient corrective technique for spinal deformities. Each study showed favorable outcomes for surgical complications. The last row shows the combined results of these three studies.

Authors	Sample size	Average preoperative coronal imbalance (mm)	Average postoperative coronal imbalance (mm)	Magnitude difference (mm)	Complications
Makhni et al. [6]	24	63	47	16	None
Redaelli et al. [13]	2	49.5	23.5	26	None
Buell et al. [3]	19	80	10	70	2 postoperative 2 intraoperative
TOTAL	45	64.16	26.83	37.3	4 cases

## Conclusions

The KR technique for spinal instrumentation has proven to be a safe and effective technique for correcting CI. This technique was found to provide radiographic and clinical success, along with low complication rates among adult and pediatric spinal deformity patients. However, despite these encouraging outcomes, more studies are warranted to assess and characterize potential limitations. Further studies with longer follow-up durations are needed in order to assess the long-term outcomes of this technique, especially in patients with degenerative pathologies or comorbidities. Additional knowledge on this technique will help guide the practice of spine surgeons to make appropriate surgical decisions as they weigh the risks and benefits of this technique. Lastly, an established predetermined method to define candidates for this technique is also needed in order to avoid cases of intraoperative or postoperative complications. Identifying certain patient characteristics should aid in patient selection. Nevertheless, our presented case along with the general trend of published results preliminarily characterizes the KR technique as an option in the armamentarium of spine surgeons for patients with spinal deformities.
